# No safe place: Prevalence and correlates of violence against conflict-affected women and girls in South Sudan

**DOI:** 10.1371/journal.pone.0237965

**Published:** 2020-10-12

**Authors:** Mary Ellsberg, Junior Ovince, Maureen Murphy, Alexandra Blackwell, Dashakti Reddy, Julianne Stennes, Tim Hess, Manuel Contreras

**Affiliations:** 1 Global Women’s Institute, George Washington University, Washington, D.C., United States of America; 2 Department of Global Health, Milken Institute School of Public Health, George Washington University, Washington, D.C., United States of America; 3 International Rescue Committee, Juba, South Sudan; 4 International Rescue Committee, London, United Kingdom; Washington University in St. Louis, UNITED STATES

## Abstract

**Background:**

Conflict and humanitarian crises increase the risk of both intimate partner violence and non-partner sexual violence against women and girls. We measured the prevalence and risk factors of different forms of violence against women and girls in South Sudan, which has suffered decades of conflict, most recently in 2013.

**Methods:**

A population-based survey was conducted among women aged 15–64 in three conflict-affected sites in South Sudan: Juba, Rumbek, and the Protection of Civilian Sites (PoCs) in Juba between 2015 and 2016.

**Findings:**

A total of 2,244 women between the ages of 15–64 were interviewed. Fifty percent (in the Juba PoCs) to 65% (in Juba and Rumbek) of all female respondents experienced either physical or sexual violence from a partner or non-partner in the course of their lifetimes. Approximately 35% of respondents have experienced rape, attempted rape or other forms of sexual violence by a non-partner during their lifetime. For ever-partnered women, lifetime prevalence of physical and/or sexual partner violence ranged between 54% in the Juba PoCs and 73% in Rumbek. Restrictive marital practices and gender norms, and experiences of conflict were major drivers of both partner and non-partner violence.

**Conclusion:**

Women and girls in South Sudan suffer among the highest levels of physical and sexual violence in the world. Although the prevalence of sexual assault by non-partners is four times the global average, women are still at greatest risk of physical and sexual assault from intimate partners. Conflict-related and intimate partner violence reinforce each other and are upheld by restrictive gender norms and marital practices. Expansion of comprehensive services, including health and psycho-social support for survivors is urgently needed. Moreover, policies and laws to prevent violence against women and provide survivors with access to justice should be given high priority within the ongoing peacebuilding process in South Sudan.

## Introduction

“We are tired of being raped. We have taken our concerns to the Chiefs, but we have not heard back as yet.” Woman activist from Rumbek, South Sudan [1].

Violence against women and girls (VAWG) is a serious human rights violation and a significant global health and security issue. In 2013, the World Health Organization (WHO) estimated that 33% of women globally experience sexual and/or physical intimate partner violence (IPV), and 7% of women experience non-partner sexual violence (NPSV) at some point in their lives [2]. There is some evidence indicating that NPSV against both women and men increases during conflict, and is frequently used as a weapon of war [3, 4]. The global prevalence of sexual violence among refugees and displaced persons in complex humanitarian emergencies is estimated to be 21.4%, suggesting that approximately one in five women who are refugees or displaced by an emergency experience sexual violence whether by a partner or non-partner [5]. Recent studies indicate that IPV may actually be more common than conflict-related sexual assault, even in high conflict settings [3, 6–8]. However, due to differences in the way that violence is defined and measured, it is difficult to compare emerging research findings on VAWG in conflict-affected settings. This type of research entails a series of complex methodological, as well as ethical and safety, challenges. A key challenge is how to address temporality; given the constraints of a cross-sectional survey, it is difficult to disentangle how acts of IPV or NPSV take place in relation to each other and to periods of conflict, particularly in a setting where multiple and prolonged periods of conflict have occurred. Selecting a study population, and sampling frame is particularly challenging in a constantly changing environment. Perhaps most importantly, ensuring the safety of both respondents and researchers in a volatile context is an ongoing and critical challenge [9]. There is a need for more methodological consistency in order to build an evidence base on VAWG in conflict and humanitarian settings that can contribute to improved strategies for preventing and responding to this pressing concern [3, 5, 10, 11].

### Violence against women and girls in South Sudan

South Sudan, the world’s newest nation, gained its independence from Sudan in 2011, after decades of conflict. In December, 2013, a new conflict broke out initially between different factions of the political leadership, that then evolved into a broader ethnic-based conflict that has not been fully resolved at the time of this publication. Since the beginning of the 2013 Crisis, tens of thousands of people have been killed, and more than a million were displaced from their homes, including more than 400,000 who were forced to flee to neighbouring countries and to United Nations (UN) Protection of Civilian (PoC) sites across South Sudan [12]. The 2013 Crisis has further worsened the situation for women and girls in South Sudan. Women and girls who have been displaced by this conflict often reside in PoC sites where they face significant dangers collecting firewood, fuel, water, food and shelter materials. NGO and UN assessments have found that sexual violence is widespread in these sites [13, 14]. Although peace agreements were signed by the parties in August 2015, and again in 2018 and 2020, there have been many delays in establishing the transitional government, and the threat of violence continues. The conflict continues to exacerbate poverty and instability throughout large parts of the country and has eroded the education and political systems and devastated the economy, leaving few institutional structures to deliver services or guarantee the rule of law [15]. The Coronavirus pandemic has further weakened the peace process, and according to the Secretary General of the UN, in June, 2020, “South Sudan continues to be gripped by a serious humanitarian crisis. The cumulative effect of prolonged conflict, chronic vulnerability and weak essential services compounded by emerging health risks have left some 7.5 million people in need, while hunger threatens over half of the population” [16].

In addition to the 2013 Crisis, general inter-communal conflicts have been a continuing facet of life in parts of South Sudan. A central driver of these conflicts is South Sudan’s cattle culture, which is an important source of wealth and social status. Cattle continue to be essential for cementing social bonds, especially through marriage, as ‘bride price,’ is commonly paid in cattle in many parts of the country. These conflicts often centre on localised tensions such as land for cattle grazing, cattle raiding, and abduction of women and girls for marriage [17]. Many of these incidents trigger revenge attacks/killings from the victimised community, causing a cycle of revenge that perpetuates continuing insecurity. Although inter-communal conflicts have existed for years in South Sudan, they become even more common in times of war and famine when families who have lost their cattle seek ways to regain their wealth by raiding neighbouring communities. All of these conditions increase insecurity for women and girls, who are the main victims of sexual violence and abductions. Moreover, gender inequality is deeply entrenched in South Sudan and is expressed though lack of access of girls and women to education and livelihoods, as well as other forms of VAWG such as child and forced marriage, intimate partner violence, and other patriarchal practices in South Sudan, such as “widow inheritance,” whereby women whose husbands have died are forced to marry the brother or male relative of the deceased husband in order to keep her children and any assets or property she might inherit within the in-laws’ family [18].

The purpose of this study was to identity the forms, trends and prevalence of all forms of VAWG in conflict-affected regions of South Sudan, as well as to identify direct and indirect drivers of VAWG in conflict settings in South Sudan.

## Materials and methods

The overall study utilized a mixed-methods approach, employing both quantitative and qualitative methods. This paper presents only the findings from the quantitative study; the qualitative findings are presented elsewhere [1]. A population-based household survey was administered to a representative sample of women aged 15–64 in three locations: Juba City, Rumbek Centre and the Juba PoCs. The survey was based on the *WHO Multi-country Study on Women’s Health and Domestic Violence Against Women v 12*.*0* [19], an instrument that has been used to measure intimate partner violence and non-partner sexual violence in many settings throughout the world. This questionnaire was adapted to the context of South Sudan. One-hour interviews were administered in-person by trained female enumerators utilizing a mobile phone interface to reduce data collection/entry errors. The survey was delivered in the main local languages in each setting–Juba Arabic, Nuer or Dinka. A smaller sample of men was also interviewed about their experiences of sexual violence and perpetration of sexual and intimate partner violence against women and girls. The results of this survey are presented elsewhere [1].

### Development of the questionnaire

In order to adapt the WHO questionnaire to address conflict settings, and the context of South Sudan in particular, several steps were taken. A consultation meeting was held in 2015 with leading experts and researchers in the field, as well as an extensive review of other instruments and methods used for measuring violence in conflict. A comprehensive literature review of research on violence against women and girls was also conducted [8]. Formative research, involving in-depth interviews and focus group discussions was carried out in 2015–16 in South Sudan, to gain the perspectives of a broad variety of stakeholders, including community men and women from each of the study sites, youth, traditional leaders, health providers, local authorities and humanitarian actors. Over 500 individuals participated in the formative research. Based on this previous work, a conceptual framework was developed to understand how different forms of VAWG are directly and indirectly influenced by conflict. This framework builds on the socio-ecological framework to identify drivers and protective factors at multiple levels, from the individual, interpersonal, community, institutional, and societal levels [8, 20]. The model addresses different types of physical and sexual violence, with a focus on IPV and non-partner sexual violence (NPSV), whether occurring in the home, in the community, and in the context of conflict. Additional factors include; socio-demographic characteristics of the women and her partner (age, education, ethnicity, work, housing, sanitation, household fuel); marital practices (bride price, early, child and forced marriage, wife inheritance, possibilities for divorce); gender social norms (views on roles and rights of women versus men, victim blaming attitudes, acceptance of violence); and exposure to conflict and traumatic events. This conceptual framework was used in the adaptation of the survey instrument as well as the data analysis plan. Although the model includes community and macro level variables, this study focuses primarily on variables that can be measured at the individual level.

The WHO questionnaire covers the following topics: basic household information; demographic characteristics of the respondent and her spouse; circumstances of marriage and attitudes around gender and the acceptability of violence; physical, sexual, psychological violence and controlling behaviour experienced by an intimate partner; sexual violence at any age by someone who was not their partner; perpetrators of violence; and use of services.

In order to explore the direct and indirect effects of conflict on IPV and NPSV in the specific context of South Sudan, additional questions were derived from the formative research to cover exposure to conflict or other traumatic events, including abduction, displacement, and attacks on their villages, as well as physical and sexual violence by armed actors and others. For each type of conflict that the respondent might have experienced, follow-up questions addressed temporality; whether these events occurred during the Civil War, the 2013 Crisis, or as a result of ongoing inter-communal violence (S1 Table).

The key outcome variables are those measured by the WHO Questionnaire. In certain cases, discussed below, some of these measures were adapted for the current context. **Intimate partner violence (IPV)** refers to behaviour by a current or previous husband, boyfriend, or other partner that causes physical, sexual, or psychological harm, including physical aggression, sexual coercion, psychological abuse, and controlling behaviours. **Emotional IPV** was defined as being insulted, or made her feel bad about herself, humiliated in front of others, having things done to scare or intimidate her on purpose, or receiving verbal threats to hurt her or someone she cared about. **Physical IPV** was defined through the following acts: being hit, slapped, having something that could hurt her thrown at her, pushed, shoved, beaten up, hit dragged or kicked, choked, burnt on purpose, being threatened with a knife, gun or other weapon or having a weapon used on her. Sexual IPV including: being forced to have sex when she didn’t want to, having sex because she was afraid of what he might do if she refused, or being forced to do anything sexual that she found humiliating or degrading. **Economic violence** was defined as refusing to let her earn money, taking away her money without asking, or refusing to give her money for household needs when he has money for other things. Only ever-partnered women were asked about experiences of intimate partner violence. For each act, respondents were asked whether it had ever happened in their lives and whether it happened during the 12 months previous to the interview. Women who had been exposed to any kind of IPV were asked whether it occurred before, during, and after the Civil War (if they were old enough to have experienced it), the 2013 Crisis, or inter-communal violence. If the answer was positive, they were asked whether the violence was the same, less severe, or more severe during each of the conflicts.

**Physical violence and traumatic conflict-related events** were defined as having been: beaten, kicked, hurt with a stick or other object; threatened with a gun, knife, or other weapon; seriously injured; disfigured; or forcibly abducted.

**Non-partner sexual violence** was defined as any sexual act, attempt to obtain a sexual act, or other act directed against a person’s sexuality using coercion by a non-intimate partner. This includes acts of sexual violence committed by armed actors, family or community members, teachers, or other individuals, known or unknown to the survivor [21]. Respondents were asked about the following acts: being forced to undress or strip off clothing; forced into having sex when she did not want it **(rape),** for example, by being threatened, held down, or put in a situation where she could not say no; someone attempted to forced her into sex **(attempted rape)**, touched her sexually or did anything else sexually that she did not want to do **(unwanted touching)**; promised to give her gifts, help pay for things or help in other ways in exchange for sex **(sexual exploitation.)** For each of the outcome variables, respondents were asked whether it had occurred ever in their lifetimes, during the last 12 months, and whether the violence occurred in the context of the Civil War, the 2013 Crisis, and intercommunal violence. Women were also asked about multiple occurrences and their age the first time and the last time it occurred. Additional circumstances, including information on perpetrators, was asked about the event that the respondent considered most severe.

### Study population and sampling

**Fig 1:**
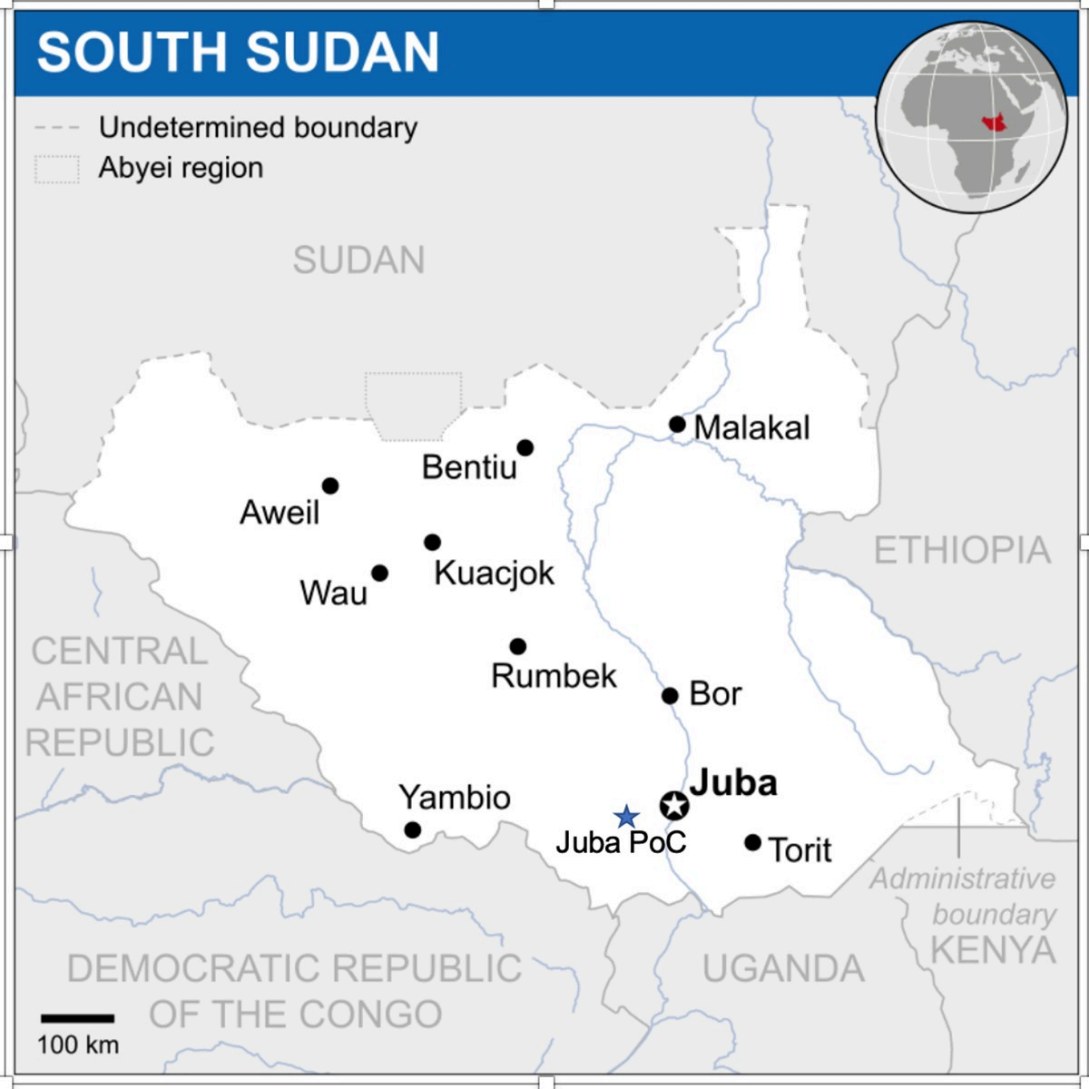
Map of South Sudan with major cities and juba protection of citizens site. Adapted from https://commons.wikimedia.org/w/index.php?curid=32650063.

The household survey was conducted in three sites with very different characteristics, each of which had been affected by some form of violence or displacement (Fig 1). Juba is the capital of South Sudan, and was the initial setting of the 2013 Crisis. The Juba Protection of Civilians (PoC) sites, are located outside of Juba on land belonging to the United Nations Mission. When fighting started in Juba, a large part of the Nuer population in Juba fled to the UN Mission for sanctuary, and at the time of the field work there were approximately 35,000 people living in temporary shelters (tents) under the protection of the United Nations. The third site is the town of Rumbek, in Lakes State. The population of Rumbek was less affected by the 2013 Crisis, but it is a constant source of inter-communal fighting and deadly cattle raids, with a great impact on women and girls. The Civil War affected women and girls in all three sites.

The study sites were chosen to provide insight on different types of VAWG in areas with distinctly different cultures and experiences of conflict. Therefore, the results do not represent the population of South Sudan as a whole. This study examined the effects of three main armed conflicts: the Sudanese Civil War which led to the 2005 Peace Accords, and the independence of South Sudan from Sudan in 2011; the 2013 Crisis; and ongoing intercommunal conflict.

A multi-stage cluster sampling design was used to select individual households for inclusion in the cross-sectional survey. Randomly selected bomas (South Sudan’s smallest administrative unit) or blocks (in the PoC sites) for inclusion were further subdivided into smaller clusters (approximately 100–250 households per cluster) based on geographic distribution. A systematic sampling strategy was used for household selection, whereby a starting point within a cluster was chosen randomly and then a standard interval of five houses was applied for each subsequent household selection. In each selected household a roster of eligible women (between the ages of 15–64) was created and one eligible woman was randomly selected to be interviewed. No substitutions were made. To account for non-responses and households without eligible women, a margin of approximately 15% was included in the sampling frame. The aim was to obtain 2,400 completed interviews with 800 women in each of the three sites.

Data collection began in June 2016, but was paused in mid-July due to a new outbreak of violence in Juba City. Data collection was completed in Rumbek by the end of July, while data collection in the Juba PoC sites was resumed and completed in November-December 2016. Data collection was not completed in Juba City due to concerns for the safety and security of the interviewers and respondents.

### Data analysis

Data were analysed using descriptive statistics as well as bivariate and multivariate statistical methods using SPSS version 25. Descriptive statistics were used to present the prevalence and characteristics of VAWG, experiences of conflict and social norms. The sites were selected precisely because of their different characteristics, and as they do not represent a single geographic region, all descriptive results are presented separately for each site. Kaplan Meier Survival Analysis was used to calculate the median age of marriage among all women, in order to include the experiences of women who were not yet married at the time of the interview. For the analysis of factors related to experiences of violence (both IPV and NPSV), a multi-step process was used. First, individual relationships between the potential factors associated with both lifetime NPSV and lifetime physical and/or sexual IPV were explored using Pearson chi square. Analysis of factors associated with NPSV was performed using the data from all women, whereas the analysis of factors associated with IPV was performed on a subset of ever-partnered women.

Variables with a statistically significant (p < 0.05) association were then included in binary logistic regression analysis. Crude (unadjusted) odds ratios (COR) were calculated for each independent variable and the dependent variables separately for each site. Thereafter, all variables with significant associations (p < .05) from one or more of the site-specific analyses were included in the analysis of a pooled data set. First, each independent variable was tested against the dependent variables, and adjusted odds ratios (AOR) were calculated for each variable, using a dummy variable to adjust for site. Next, step-wise multivariate logistic regression modelling was performed, entering variables by block, starting with demographic variables, marriage circumstances, gender attitudes, and finally experiences of conflict and displacement. The final multivariate model presented includes all variables that maintained significance, with adjustment for age and site. Displacement was not included in the final model, as all of the respondents in one of the sites (Juba PoC) were currently displaced.

### Ethical and safety considerations

The research protocol was approved by the Institutional Review Board (IRB) of the George Washington University as well as the What Works Technical Advisory Group (TAG) in South Sudan, which is an independent body of experts in VAWG research and programming in South Sudan made up of local, national and international practitioners representing both NGO and government structures. Permission to conduct the research was secured with appropriate authorities at national and local levels.

Ensuring the safety of the participants and the research team at all times was a priority for the study. Verbal informed consent was obtained by all participants. Because disclosing violence within the family could lead to additional risk of harm, the requirement to obtain consent from parents or guardians for young women under 18 years of age was waived by the IRB. IRC security protocols were closely followed by the field team throughout the entirety of data collection, and the research team worked with the IRC country team to determine the research sites and methods for data collection based on safety criteria and availability of referral services for respondents. The research strictly adhered to the WHO’s Ethical and Safety Recommendations for Researching, Documenting and Monitoring Sexual Violence in Emergencies, including interviewing only one woman per household, ensuring privacy during interviews, offering GBV services to all respondents, and making GBV officers and vehicles available at all times for emergency situations arising for respondents or interviewers. Female interviewers with diverse ethnic backgrounds who spoke the local languages (primarily Dinka, Nuer, or Juba Arabic), as well as English, were trained over a four-week period. Training included information about gender-based violence and trauma informed interviewing techniques. In the Juba PoC site, due to the difficulties of access, interviewers were recruited among camp residents. A detailed description of the safety measures established during the study is presented elsewhere [22].

## Results and discussion

### Demographic and socio-economic characteristics of respondents

A total of 2,244 women were interviewed in the three sites. The household response rate was 87% and the individual response rate was 89%. Participants in all three sites described themselves as overwhelmingly of Christian faith and were generally young, with median ages ranging from 22 to 26 years of age. However, there were considerable differences among the sites on other demographic characteristics (Table 1). Women from Juba city were primarily from tribes from the Equatorian region of South Sudan (e.g. Bari, Madi, Acholi, Kuku, etc.), while respondents from Rumbek were almost exclusively from the Dinka tribe, and those from the Juba PoC sites were overwhelmingly from the Nuer tribe. Participants from Juba City were the most educated, where 83% of women had some formal education, compared to 49% in Rumbek and 62% in the Juba PoCs.

**Table 1:** Demographic and social characteristics of respondents.

	Juba	Rumbek	PoCs	Total
	n = 477	n = 804	n = 963	n = 2244
	%	%	%	%
Partnership Status				
Never partnered	4.0	15.5	24.5	16.9
Ever partnered	96.0	84.5	75.5	83.1
Currently married or living together	87.2	80.1	67.9	76.4
Currently no partner, previous partner	8.8	4.4	7.6	6.7
Age				
Median age	26	25	22	24
Age by groups				
15–19	17.4	23.8	35.9	27.6
20–29	45.1	40.2	41.5	41.8
30–39	22.9	20.9	14.4	18.6
40–64	14.6	15.1	8.1	12.0
Education				
No formal education	17.0	50.9	37.6	38.0
Any formal education	83.0	49.1	62.4	62.0
primary	39.6	23.6	34.0	31.5
secondary	35.0	23.1	24.2	26.0
higher	8.4	2.4	4.3	4.5
Religion				
Islam/other	6.7	4.7	5.9	5.7
Christian Catholic	52.0	41.9	47.1	46.3
Christian Protestant	41.3	53.4	46.9	48.0
Tribe				
Dinka	9.4	98.0	2.1	38.0
Nuer	1.3	0.4	89.9	39.0
Equatorian/Other	76.9	0.7	3.3	23.0
Other	12.4	0.9	4.7	
Main Occupation				
Not Working/domestic work/student	67.7	75.4	91.4	70.6
Working	32.3	24.6	8.6	19.4
Household Source of income				
No Income/Humanitarian Aid	7.3	6.5	62.2	30.6
Money from own work	22.9	25.9	3.8	15.8
Support from husband/relatives	69.8	67.7	33.9	48.6
Household Source of Fuel				
Charcoal	88.1	40.0	70.5	63.3
Firewood/grass/leaves	11.9	60.0	29.3	36.7
Age at marriage				
Median age at marriage^a^	19	18	18	18
Age at marriage^b^	n = 360	n = 615	n = 604	n = 1579
Marriage 15 or younger	6.9	7.0	9.6	8.0
Married at age 15–19	64.2	78	76.2	74.2
Married at age 20 or older	25.9	14.9	13.7	16.8
Number of times married				
once	81.4	81.8	85.3	82.8
twice or more	19.7	18.2	14.7	17.2
Husband has other wives at same time				
no	58.1	44.2	61.9	54.1
yes	41.9	55.8	38.1	45.9
Number of other wives				
No other wives	58.1	44.2	61.9	54.1
1 other wife	27.2	25.6	17.4	22.8
2 or more	14.7	30.2	20.7	23.1
Who made the decision for her to marry?				
She/They both decided	68.3	21.5	55.8	45.3
Her family decided	4.7	16.9	12.4	12.4
Husband decided	20.3	27.3	19.9	22.9
Husband’s family decided	6.7	34.3	11.9	19.4
Was she consulted about the marriage?				
Yes	90.6	74.8	82.8	81.4
No	9.4	25.2	17.2	18.6
Did her husband pay a bride price?				
Yes	57.8	88.1	84.4	79.8
No	42.2	11.9	15.6	20.2
Reasons for marriage ^c^				
Married due to love or desire for family	83.9	84.7	74.2	80.5
Married due to abduction, pregnancy or rape	15.0	4.1	16.6	11.3
Married for economic reasons	11.9	16.6	10.4	13.2
Age difference between woman and partner				
Woman older or same age	14.4	16.7	10.8	13.9
Partner older 1–10 years	53.9	36.6	53.6	47.1
Partner older >10	31.7	46.7	35.6	39.0
Relationship of current to former partner ^d^	n = 11	n = 44	n = 31	n = 86
No relation	100.0	31.8	30.0	39.6.
Brother of former partner	0.0	50.0	54.0	45.3
Male relative of former partner	0.0	18.2	16.0	15.1

^a^Calculated using Kaplan Meier Survival Analysis among all women (including non-married women) who were able to provide a specific current age or age at marriage.

^b^ Among ever-married women

^c^Responses do not add up to 100% due to multiple responses.

^d^Among women who were widowed and remarried

Only around a quarter of respondents worked outside the home, and this rate was far lower for respondents who had been displaced into the Juba PoCs (9%). The majority of women in Juba and Rumbek reported that the household’s main source of income came from their husbands or relatives, compared to Juba PoCs, where nearly two thirds of women (62.2%) reported that their household had no income or that they relied exclusively on humanitarian aid. Only 4% of respondents in the PoCs said that their household’s primary income was money from her own work, compared to about a quarter of women from Juba City and Rumbek.

A majority of female respondents had a current partner (ranging from 68% in Juba PoCs to 87% in Juba City). A quarter of women in Juba PoCs had never had a partner (this site also had the youngest median age of respondents) compared to the other sites (16% for Rumbek and 4% in Juba). Of the women and girls who were partnered, most were currently living with their partners. However, a substantial proportion of women in the Juba PoCs (46%) were not currently living with their husband. The most commonly cited reason for this was because of the crisis generally (54%) and because their husband was missing due to the crisis (29%). Women from Juba City less often cited the conflict as the reason they lived apart from their husband—67% reported that the reason they lived apart from their husband was not related to the conflict.

Over half of women in Rumbek, and more than one third of women in Juba and Juba PoCs live in polygamous marriages. The percentage of women whose husbands had two or more other wives ranged from 15% in Juba to 21% in Juba PoCs and 30% in Rumbek. The greatest number of wives reported was 20. The payment of bride price was most commonly reported in Rumbek (88%) and the Juba PoCs (84%). Bride price payment was less common in Juba City, where 58% of respondents reported that it was paid. Across all three sites—but particularly in Juba City—bride price payment was less common among younger women (between 15–19 years of age). The circumstances of marriage varied widely among the sites. Whereas the majority of women In Juba City and the Juba PoCs reported that it was primarily the decision of the respondent and her future husband to get married, over three quarters of women in Rumbek reported that their husbands were chosen by someone else. Although most women were consulted about the marriage, one quarter of women in Rumbek, compared to 9% in Juba and 17% in Juba PoCs, reported that they were not consulted about the decision to get married.

Among the formerly married women, divorce or separation was uncommon—the majority were widowed. Only a small proportion of respondents reported being widowed and re-married (n = 83). Among these women, about 70% of respondents in Rumbek and Juba PoCs were currently married to a male relative of their deceased husband, usually the brother. This suggests that they were “inherited” by the husband’s family, a common practice in South Sudan. No women from Juba reported being married to a relative of their former husband.

In all three sites, the male partners of respondents had higher educational attainment than their wives, with nearly two thirds (62%) having received secondary education or higher, compared to 31% among female respondents (Table 2). They were also much more likely to be working for cash (71%), compared to women (19%). Men in Juba were more likely to be engaged in professional work (21%) compared to men in Rumbek (15%) or the PoCs (16%). Overall, almost one-third of men were employed in the military or police, with the largest proportion based (35%) in Rumbek.

**Table 2:** Partners’ characteristics.

	Juba	Rumbek	PoCs	Total
			
	n = 458	n = 679	n = 727	n = 1864
	%	%	%	%
Partner's Education				
No formal education	9.0	33.0	30.8	26.2
Any education	91.0	77.0	69.2	73.8
Primary	12.9	14.7	9.8	12.3
Secondary	41.9	29.7	23.0	30.1
Higher	36.2	22.5	36.5	31.3
				
Partner's Profession				
Never worked	14.6	33.6	33.7	29.0
Professional	21.2	14.6	15.7	16.6
Semi-Skilled/Unskilled/Manual/Other	38.4	16.9	18.6	22.9
Military/Police	25.8	34.9	32.0	31.5

### Experiences of conflict

The results of the household survey show that the populations in all three settings have been severely affected by armed conflict, albeit with different characteristics and intensity at different times. During the lengthy Sudanese civil wars, almost the entire country was affected by violence at some stage of the conflict, while the 2013 Crisis primarily affected the population of Juba City and the Juba PoC sites, among the study sites. In addition to the Civil War, Rumbek was primarily affected by intercommunal violence, often in the context of cattle raids (Table 3).

**Table 3:** Experiences of conflict.

Displacement status	Juba	Rumbek	PoCs	Total
	n = 477	n = 804	n = 963	n = 2244
	%	%	%	%
Never Displaced	63.7	50.5	0.0	31.6
Ever displaced	36.3	49.5	100.0	68.4
Currently Displaced	6.5	39.7	100.0	58.5
Formerly Displaced	29.8	9.8	0.0	9.8
Duration of displacement	n = 173	n = 397	n = 963	n = 1533
All of my life	4.2	9.2	15.8	11.0
More than one year	23.1	25.2	76.9	47.0
Less than one year	9.0	15.0	7.3	10.4
Cause of displacement ^a^				
Displaced because of the 2013 Crisis	13.8	9.6	97.5	48.2
Displaced because of the Civil War	21.6	9.0	5.5	11.5
Displaced because of Intercommunal/Tribal conflicts	1.7	31.3	10.7	16.2
Attacks on village of residence ^a^	n = 477	n = 804	n = 963	n = 2244
Experienced any attacks on her village ^b^	43.0	52,9	59.8	53.7
Village was attacked during the 2013 crisis	30.0	31,2	67.7	40.9
Village was attacked during the Civil War	32.9	28.2	32.0	30.8
Village was attacked during an intercommunal raid	27.9	65.8	44.0	52.6
Forcibly detained or abducted	9.4	6.7	10.1	8.7
Ever beaten, kicked, threatened or had weapon used	24.9	38.3	33.7	33.5
Ever experienced physical violence or traumatic event during conflict ^c^	16.8	31.1	37.4	30.7
Have experienced physical violence during the last 12 months	4.6	9.8	11.9	9.6
Perpetrators of physical violence ^d^	n = 119	n = 308	n = 325	n = 752
Family members	39.5	36.7	23.1	31.3
Friends or acquaintances	41.2	25.0	19.7	25.3
Police officers	0.0	4.5	24.0	12.2
Armed actors	8.4	6.2	17.5	11.4
From other communities	6.7	28.2	27.1	24.3
Complete strangers	3.4	0.0	0.6	0.8

^a^Response do not add up to 100% due to multiple responses

^b^ Women were asked about multiples types of attacks on their village. Some women responded positively to experiencing specific attacks, despite responding negatively to the general question

^c”^Traumatic event” was defined as having been beaten or physically abused, disfigured, sustained injuries or abduction in the context of conflict.

^d^May include multiple experiences of violence and multiple perpetrators

Not surprisingly, women and girls residing in the Juba PoC sites were the most affected by the 2013 Crisis, with 100% of residents having been displaced, and 68% reporting that their homes or communities were attacked. These circumstances drove the population to seek the protection of the UN and directly led to the establishment of the PoC sites [23].

In addition, women in the PoCs also experienced the most prolonged displacement; over 90% of respondents have been displaced for over one year and 16% reporting that they had been displaced during their whole lives. Women and girls in the other two sites also experienced displacement; 40% in Rumbek and 7% in Juba City were currently displaced. However, the length of displacement was less in these two sites compared to the PoCs. Inter-communal conflicts have persisted in many areas since the signing of the CPA in 2005. While these tensions were experienced throughout the study locations, they were more frequently reported in Rumbek and Juba City. In Rumbek, 66% of women reported that their village had been attacked by another community primarily for the purpose of cattle raiding. Nearly one in ten women in Juba and Juba PoC sites have been forcibly detained or abducted. Over 30% of women experienced a physical violence or traumatic event related to conflict.

Whereas the majority of perpetrators of physical violence in Juba and Rumbek were either family members of individuals known to the respondent, perpetrators of violence in Juba PoCs were more likely to be police officers and other armed actors. Individuals from other communities were also mentioned in both the PoCs and Rumbek as key perpetrators.

### Non-partner sexual violence

In the household survey, between about 35% of women in all three sites reported experiencing some type of non-partner sexual violence during their lifetimes (Table 4). Women experiencing non-partner sexual assault experienced a variety of types of violence. In Juba, 18% of respondents experienced rape or attempted rape, compared to 25% in Juba PoCs. Between 5% to 11% of respondents reported sexual violence within the past 12 months, across the three sites.

**Table 4:** Sexual violence by non-partners and experiences of conflict.

Experiences of sexual violence by someone other than a partner*	Juba	Rumbek	PoCs	Total
	n = 477	n = 804	n = 963	n = 2244
	%	%	%	%
Forced to undress or stripped off your clothes	3.4	16.3	18.3	14.4
Unwanted touching	21.6	18.2	22.4	20.7
Sexual exploitation (offered money or gifts for sex)	22.2	9.5	21.1	17.2
Attempted rape	16.1	18.7	21.0	19.1
Rape	6.3	17.4	21.7	16.9
Rape or attempted rape	18.0	23.0	25.2	22.9
Any sexual violence ever	35.4	34.5	34.6	34.7
Any sexual violence during last 12 months	4.8	8.3	10.7	8.6
Did the sexual violence occur during any of these times *	n = 169	n = 277	n = 333	n = 779
During the 2013 Crisis	31.4	30.3	59.2	42.9
During the Civil War	10.7	23.5	24.9	21.3
During Intercommunal violence	3.6	61.7	23.1	32.6
During abduction	3.0	5.8	19.8	11.2
During displacement	1.2	5,4	39.9	19.3
During none of these situations	88.8	29.6	23.7	39.5
Perpetrators of the most severe act of sexual violence*				
Family member	6.5	32.1	11.1	17.6
Friend or acquaintance	47.9	18.8	5.7	19.5
Police officers	1.2	4.3	22.2	11.3
Armed actors	4.7	5.8	6.6	5.9
Someone from another community	9.5	24.9	23.4	20.9
Humanitarian workers	3.0	1.4	0.6	1.4
Complete strangers	27.8	31.4	3.3	18.6
Number of times have experienced sexual violence				
Once	57.4	63.5	58.9	60.2
A few times	33.1	28.2	17.4	24.6
Many times	9.5	8.3	23.7	15.1
Age at first incident				
15 or less	13.0	19.5	21.9	19.1
15–19	46.2	36.5	43.5	41.6
20–24	20.1	20.9	13.8	17.7
25 +	20.7	23.1	20.7	21.5

*Responses due not add up to 100% due to multiple responses

The characteristics of sexual violence reported by women differed greatly across the three sites. In Juba City, the most common perpetrators of sexual violence against women and girls were either acquaintances (not family) or complete strangers. The majority of sexual violence reported in Rumbek was associated with intercommunal violence, and perpetrators were mostly either family members and other known persons, or people from another community and complete strangers. Sexual assault in Juba PoCs was more often associated with displacement or abduction, and perpetrators were more likely to be police officers and members of another community.

Although a majority of women reported experiencing sexual violence only once in their lifetimes, a considerable proportion of women in all three sites reported they had experienced this violence multiple times. Almost one quarter of women (23.7%) in the Juba PoCs reported experiencing many incidents of non-partner sexual violence. No matter the perpetrator, violence begins early in the lives of women and girls in each of the study sites—over 50% of respondents across all three sites and over 60% of respondents in the Juba PoCs reported that the first incident of sexual violence occurred before the age of 20.

Over 20% of female respondents in both Juba City and the Juba PoCs and 10% in Rumbek reported sexual exploitation, defined in this study has having sex in exchange for money or gifts of food and other commodities. This is likely related to increased economic insecurity, coupled with either the death or deployment of the husband to the front lines. The lack of livelihood opportunities for women in the PoCs, especially when abandoned or widowed by their husbands, places them at particular risk for sexual exploitation.

### Intimate partner violence

Lifetime prevalence of physical and/or sexual IPV was high in all of the sites: 60% in Juba City; 73% in Rumbek and 54% in Juba PoCs (Table 5). Not only is physical IPV extremely common throughout the study sites in South Sudan, but it is also notable for its severity and frequency. Almost three-quarters of women who reported IPV experienced the most severe forms of violence (defined as being hit, kicked or dragged, choked or burnt, or threatened with a knife or gun) compared to moderate violence (defined as slapped or something thrown at, pushed or shoved). In addition to the severity of the violence, women in each site experienced frequent acts of violence. Acts of intimate partner violence often lead to physical injury, according to the respondents. This was particularly true for women in Rumbek and the Juba PoCs, where approximately 60% of women who experienced physical or sexual IPV reported experiencing an injury as a result. Almost 40% of women in the Juba PoCs reported severe injuries (broken bones, teeth, internal injuries, miscarriage, permanent disability or disfigurement) due to IPV.

**Table 5:** Intimate partner violence and controlling behaviours by a partner.

Experienced IPV ever in her lifetime ^a^	Juba	Rumbek	PoCs	Total
	n = 458	n = 679	n = 727	n = 1864
	%	%	%	%
Type of IPV experience				
Economic violence	34.7	54.2	31.1	40.4
Emotional violence	47.8	67.7	43.3	53.3
Physical violence	42.4	67.2	44.3	52.1
Sexual violence	45.4	49.8	43.5	46.2
Physical and/or sexual violence	59.8	72.8	54.3	62.4
Experienced IPV during the last 12 months ^b^	n = 242	n = 363	n = 406	n = 1011
Physical violence	26.9	56.7	32.5	39.9
Sexual violence	30.2	41.6	38.2	34.5
Sexual and/or physical violence	43.0	62.8	47.0	48.6
Does your husband/partner do the following? ^a^	n = 458	n = 679	n = 727	n = 1864
Limits contact with her birth family	17.5	31.7	30.5	27.7
Insists on knowing where she is at all times	54.6	60.4	50.8	55.2
Gets jealous or angry if she talks to another man	48.0	55.5	57.9	54.6
Accuses her of being unfaithful	31.2	44.0	30.9	35.8
Makes her ask for permission to receive health care	52.2	44.8	46.2	47.2
Number of acts of controlling behaviour				
No controlling behaviours	27.7	26.8	27.8	27.4
1–2 controlling behaviours	47.6	32.0	41.5	39.5
3–4 controlling behaviours	24.7	41.2	30.7	33.0

^a^Among ever-partnered women.

^b^Among ever-partnered women who had seen their partners during the last 12 months

High levels of male control over all aspects of their wives’ lives was also found in all sites. Women were asked if their husbands exerted control over daily activities, such as not allowing her to visit her family or insisting on knowing always where she is. Women and girls across all three survey sites reported a high degree of control by their partners over daily activities. Of ever-partnered women, only about a quarter of respondents in each site had not experienced any controlling behaviours from their husband or partner. Women in Rumbek experienced the most control; over 40% of respondents experienced three to four of these behaviours. Respondents from Juba City were least likely to report experiencing multiple controlling behaviours; approximately 50% of respondents reported experiencing one or two of these behaviours.

### Attitudes reflecting restrictive gender norms

Respondents were asked if they agreed or disagreed with a series of statements reflecting common gender-related social norms that were identified during the qualitative research (Table 6). In almost all cases, women from Rumbek were most likely to endorse restrictive gender norms, including a wife’s obligation to have sex with her husband whenever he wants, to obey him even if he disagrees, and to tolerate violence to keep the family together. Women from Juba and Juba PoCs were less likely to endorse these views, although between one third and two thirds of women agreed with these statements. With regard to sexual violence, 46% and 39% of women in Rumbek, respectively, agreed that “if a woman is raped, she has usually done something careless to put herself in that position,” and, “if a girl is raped, she should marry the man who raped her.” Only in Juba PoCs, where the main perpetrators of sexual violence were police or armed actors, did most women disagree with the statement that a girl should marry her rapist.

**Table 6:** Endorsement of restrictive gender norms^a^.

Agrees with the following statements	Juba	Rumbek	PoC	Total
	n = 477	n = 804	n = 963	n = 2244
	%	%	%	
It is a wife’s obligation to have sex with her husband whenever he wants it	38.2	76.9	35.5	50.9
A wife should obey her husband even if she disagrees	48.0	89.8	67.4	71.3
Violence between husband and wife is a private matter and others should not intervene	63.7	87.1	43.3	63.3
A woman should tolerate violence to keep her family together	64.8	85.6	64.9	72.3
If a woman is raped, she has usually done something careless to put herself in that situation	38.2	46.0	28.5	36.8
If a girl child is raped, she should marry the man who raped her	28.3	39.1	13.5	25.8
When is a man justified in beating his wife?				
If she goes out without telling him	38.4	78.0	64.2	63.6
If she neglects the children	58.5	80.0	70.4	71.3
If she argues with him	35.4	80.1	62.9	63.2
If she refuses to have sex with him	26.8	73.0	56.7	56.2
Does not agree with any reason for violence	27.3	7.5	16.7	15.6
Agrees with 1–2 reasons	44.2	17.8	25.5	26.7
Agrees with 3–4 reasons	28.5	74.8	57.7	57.6

^a^percentage of all women who agree with the statements above, by site.

Over 90% of women from Rumbek agreed that men have a right to beat their wives for at least one reason, and 75% agreed that he would be justified for 3 or 4 reasons (e.g. if a woman goes out without telling her husband, neglects the children, argues with her husband or refuses to have sex).

### Risk factors for non-partner sexual violence

Binary logistic regression modelling was carried out for each site, and on a pooled data set, to understand the key risk factors for sexual violence by non-partners, with having ever experienced rape or attempted rape at least once as the dependent variable (Table 7). Only variables that were significantly associated with the dependent variable in one of more of the sites were shown in the site-specific models. Thereafter, each independent variable was tested against the dependent variable in a pooled data set, using a dummy variable to control for differences due to site (Model 1.). These variables were then entered step-wise in the multivariate regression analysis by block. Only variables that remained significant were kept in the final model (Model 2).

**Table 7:** Risk factors for non-partner sexual assault.

	JUBA	RUMBEK	JUBA POC	MODEL 1	MODEL 2
	COR (95% CI)^a^	COR (95% CI)^a^	COR (95% CI)^a^	AOR (95% CI)^b^	AOR (95% CI)^c^
**Location**	** **	** **	** **		
Juba	** **	** **	** **	1.00	1.00
Rumbek				**1.36 (1.02–1.81)**	**1.63 (1.15–2.31)**
PoC				**1.53 (1.17–2.02)**	**1.52 (1.07–2.15)**
**Demographic characteristics**	** **	** **	** **	** **	** **
**Respondent's age**					
15–19	1.00	1.00	1.00	1.00	1.00
20–29	1.13 (0.59–2.17)	2.19 (1.38–3.48)***	1.69 (1.19–2.40)***	1.77 (1.37–2.29)***	0.79 (0.55–1.14)
30–39	0.60 (0.30–1.40)	1.92 (1.14–3.25)**	2.18 (1.40–3.40)***	1.67 (1.23–2.2)***	0.60 (0.40–0.91)*
40–64	1.24 (0.60–2.75)	1.23 (0.67–2.26)	1.73 (0.99–3.04)***	1.45 (1.01–2.08)***	0.50 (0.31–0.80)***
Respondent's education primary and above	1.32 (0.68–2.57)	0.68 (0.48–0.94)*	0.76 (0.57–1.02)	0.72 (0.59–0.88)***	
HH's main source of fuel for cooking—wood/grass/leaves/other	0.80 (0.39–1.78)	0.84 (0.60–1.18)	2.36 (1.74–3.20)***	1.40 (1.13–1.74)*	
**Social norms**	** **	** **	** **	** **	** **
If a woman is raped, she has usually done something careless to put herself in that situation	0.84 (0.52–1.37)	1.52 (1.09–2.12)**	2.61 (1.92–3.54)***	1.73 (1.41–2.12)***	** **
If a girl child experiences raped, she should marry the man who raped her	1.46 (0.89–2.40)	2.04 (1.46–2.85)***	1.16 (0.76–1.75)	1.47 (1.18–1.82)***	1.47 (1.12–1.93)**
**Experiences of conflict**	** **	** **	** **	** **	** **
Ever experienced non partner physical violence + trauma during wars	1.71 (0.77–3.81)	2.72 (1.94–3.82)***	9.10 (6.49–12.71)***	3.00 (2.38–3.77)***	2.08 (1.60–2.70)***
Ever experienced sexual exploitation	6.78 (4.09–11.26)***	12.05 (7.04–20.62)***	12.81 (8.95–18.33)***	9.42 (7.40–12.02)***	6.83 (5.08–9.19)***
Ever experienced attack on village	1.50 (0.94–2.39)	2.68 (1.88–3.82)***	2.61 (1.88–3.62)***	2.41 (1.96–2.98)***	1.63 (1.25–2.13)***
**Intimate Partner Violence**					
Ever experienced physical and/or sexual partner violence	**1.79 (1.09–2.94)**	9.55 (5.59–16.34)***	5.54 (4.03–7.63)***	5.09 (4.02–6.46)***	3.50 (2.56–4.78)***

^a^Crude odds rations (COR) and adjusted odds ratios (AOR) with 95% Confidence Intervals (95% CI) are shown for the odds of having ever experienced non-partner sexual assault (rape or attempted rape) among women aged 15–64 in three locations in South Sudan. Figures in bold are statistically significant at the 95% level (confidence interval does not include 1·0).

^b^ Model 1 shows the association between individual independent variables and non-partner sexual assault on a pooled data set with adjustment for location only.

^c^Model 2 is the final multivariate model that includes all significant independent variables, controlling for age and location.

Note:

*p < .05

** = p < .01

*** = p < .001

In the site-specific analysis, having experienced IPV and sexual exploitation were associated with increased risk of NPSV in all three sites. In Rumbek and Juba PoCs, age, experiences of conflict (attacks on village or traumatic events), and belief that “if a woman has been raped, she has usually done something to put herself in that position” were also associated with NPSV. In addition, in Rumbek, the belief that girls should marry their rapist increased the odds of NPSV. Having any education (primary or above) was protective for NPSV. In PoCs, using firewood for fuel instead of charcoal was associated with more than a two-fold increase in the odds of NPSV.

The majority of these associations were still significant in the pooled analysis controlling for location (Model 1). However, in multivariate analysis, only support for a girl marrying her rapist (AOR = 1.5, 95%CI = 1.1–1.9), intimate partner violence (AOR 3.5, 95%CI– 2.6–4.8) and experiences of conflict were associated with rape or attempted rape, while controlling for age and location (Model 2). The extremely high association between rape/attempted rape and sexual exploitation (being offered money or gifts for sex) (AOR = 6.8, 95%CI- 5.1–9.2), indicates that the acts often referred to by the more benign term, “transactional sex,” may involve a high degree of physical coercion.

### Risk factors for intimate partner violence

Bivariate logistic regression modelling was carried out for each site, and on a pooled data set, to understand the key risk factors for intimate partner violence among ever-partnered women, using experiences of physical and/or sexual violence by an intimate partner at any point in her lifetime as the dependent variable (Table 8). Only variables that showed significant association in bivariate analysis in one of more of the sites were shown in the site-specific analysis. Thereafter, each independent variable was tested against the dependent variable in a pooled data set, using a dummy variable to control for differences due to site (Model 1). These variables were then entered step-wise in the multivariate regression analysis by blocks organized by demographic characteristics of the respondent, partners’ characteristics, marital circumstances, social norms, and experiences of conflict as independent variables. Only variables that remained significant were kept in the final model (Model 2).

**Table 8:** Risk Factors for IPV^a^.

	Juba	Rumbek	Juba PoCs	MODEL 1	MODEL 2
	COR (95%CI)	COR (95%CI)	COR (95%CI)	AOR (95%CI)	AOR (95% CI)
**Location (PoC)**				1.00	1.00
Juba				1.25 (0.99–1.59)	1.60 (1.2 2.1) ***
Rumbek				2.24 (1.80–2.81) ***	2.17 (1.6–2.9) ***
**Demographics**					
**Respondent's age**					
15–19	1.00	1.00	1.00	1.00	1.00
20–29	1.99 (1.14–3.48) **	3.40 (2.08–5.58) ***	1.78 (1.20–2.64) ***	2.23 (1.70–2.93) ***	2.21 (1.6–3.0) ***
30–39	2.30 (1.24–4.29) **	4.40 (2.50–7.72) ***	2.53 (1.56–4.10) ***	2.92 (2.13–4.01) ***	2.90 (2.0–4.2) ***
40–64	1.85 (0.94–3.65)	4.56 (2.48–8.38) ***	2.50 (1.42–4.43) ***	2.77 (1.95–3.94) ***	2.87 (1.9–4.4) ***
**Respondent's education primary or above**	0.53 (0.31–0.90) *	0.55 (0.39–0.77) ***	0.76 (0.57–1.02)	0.64 (0.52–0.78) ***	
**Partner’s characteristics**					
**Partner's education primary or above**	0.75 (0.38–1.48)	0.68 (0.47–0.99) *	0.76 (0.60–1.40) *	0.64 (0.52–0.78) ***	
**Partner's profession**					
Unemployed	1.00	1.00	1.00	1.00	
Professional	1.21 (0.64–2.26)	1.66 (0.97–2.83)	0.58 (0.37–0.91) *	0.98 (0.74–1.32)	
Semi-Skilled/Unskilled/Manual/Other	1.12 (0.63–1.97)	1.44 (0.88–2.35)	0.92 (0.60–1.40)	1.10 (0.84–1.44)	
Military/Police	1.52 (0.83–2.81)	1.93 (1.28–2.92) ***	1.19 (0.82–1.70)	1.47 (1.15–1.89) ***	
**Circumstances of marriage**					
**Controlling behaviors by partner**					
None	1.00	1.00	1.00	1.00	1.00
1–2'	1.75 (1.12–2.72) **	2.25 (1.50–3.37) ***	4.08 (2.74–6.06) ***	2.66 (2.10–3.37) ***	2.68 (2.07–3.46) ***
3–4'	4.55 (2.57–8.06) ***	15.00 (8.66–25.97) ***	12.69 (8.01–20.11) ***	10.02 (7.48–13.42) ***	7.02 (5.11–9.65) ***
**Number of Wives including respondent**					
One	1.00	1.00	1.00	1.00	1.00
Two	1.40 (0.90–2.31)	1.81 (1.17–2.81) **	2.12 (1.37–3.29) ***	1.80 (1.39–2.33) *	1.50 (1.11–2.03) **
Three or more	1.60 (9.00–3.01)	2.25 (1.46–3.46) ***	2.84 (1.85–4.36) ***	2.32 (1.77–3.05) ***	1.85 (1.35–2.55) ***
Age at marriage under 15	1.01 (.70–1.48)	0.41 (0.29–0.60) ***	0.73 (0.54–0.99) *	0.67 (0.56–0.82) ***	** **
Marriage was forced	7.70 (2.30–26.60) ***	1.2 (0.80–1.80)	1.30 (0.80–2.00)	1.50 (1.10–2.00) **	** **
Marriage due to abduction, pregnancy or rape	1.39 (0.80–2.54)	2.80 (0.80–9.60)	2.70 (1.20–3.60) ***	2.20 (1.50–3.10) ***	2.01 (1.33–3.04)***
Married for economic reasons	1.44 (0.70–2.80)	1.60 (1.00–2.80)	2.20 (1.20–3.60)**	1.70 (1.20–2.40) ***	** **
Married due to love or desire for family.	1.45 (1.00–2.20)	1.20 (0.80–1.80)	0.70 (0.50–1.00) *	1.00 (0.80–1.20)	** **
Most recent or previous husband paid a bride price	1.42 (1.00–2.10)	1.90 (1.30–2.80) ***	1.60 (1.20–2.20) ***	1.60 (1.30–2.00) ***	** **
**Social norms**					** **
It is natural that men should be the head of the family	1.18 (0.64–2.16)	0.78 (0.39–1.57)	2.01 (1.31–3.10) ***	1.42 (1.05–1.92) *	** **
Wife's obligation to have sex with her husband whenever he wants it	1.27 (0.87–1.87)	1.73 (1.16–2.60) **	1.59 (1.18–2.14) ***	1.53 (1.25–1.88) ***	1.56 (1.23–1.98) ***
A wife should obey her husband even if she disagrees.	1.13 (0.78–1.64)	0.45 (0.21–0.98) *	1.57 (1.13–2.17) **	1.42 (1.15–1.75) ***	
**Reasons for accepting violence**					
No reason	1.00	1.00	1.00	1.00	
1–2'	1.53 (0.97–2.41)	1.99 (1.00–3.97) *	2.29 (1.33–3.95) ***	1.80 (1.32–2.44) ***	
3–4'	1.49 (0.90–2.45)	2.86 (1.55–5.27) ***	3.91 (2.37–6.47) ***	2.61 (1.94–3.51) ***	
**Conflict-related experiences**					
Ever displaced	1.02 (0.69–1.50)	1.68 (1.19–2.36) ***	N/A	1.35 (1.05–1.75) *	
Ever experienced an attack on village	0.91 (0.62–1.32)	2.53 (1.79–3.57) ***	2.82 (2.06–3.87) ***	2.00 (1.65–2.44) ***	
Ever experienced rape or attempted rape during conflict	1.67 (1.01–2.76) *	10.17 (5.07–20.38) ***	7.31 (4.82–11.07) ***	5.32 (4.00–7.07) ***	2.82 (2.03–3.92) ***
Experienced sexual exploitation during conflict	3.04 (1.81–5.09) ***	6.74 (2.42–18.77) ***	5.71 (3.39–6.50) ***	4.65 (3.38–6.41) ***	2.17 (1.49–3.18) ***
Ever experienced physical violence and/or traumatic events during conflict	0.87 (0.53–1.42)	3.04 (1.99–4.64) ***	4.70 (3.39–6.50) ***	3.00 (2.38–3.77) ***	1.78 (1.37–2.33) ***

*Crude odds rations (COR) and adjusted odds ratios (AOR) with 95% Confidence Intervals (95% CI) are shown for the odds of having ever experienced physical and/or sexual violence by an intimate partner (IPV), among ever-partner women, in three locations of South Sudan. Figures in bold are statistically significant at the 95% level (confidence interval does not include 1·0).

** Model 1 shows the association between individual independent variables and physical and/or sexual IPV on a pooled data set with adjustment for site only.

***Model 2 is the final multivariate model that includes all significant independent variables, controlling for age and location.

Note: *p < .05 ** = p < .01 *** = p < .001

In the bivariate analysis by site, older women had greater odds of IPV, and having had any formal education (primary or more) was protective in all sites. In Rumbek and Juba PoCs, any formal education for the partner was associated with lower risk of IPV. In Rumbek, women whose partners were in the police or military had almost two-fold greater odds of IPV (COR 1.9, 95%CI 1.3–2.9), whereas in Juba PoCs women whose partners were professionals had a decreased risk of IPV. (COR = 0.6 95%CI 0.4–0.9) A number of indicators of patriarchal practices were also associated with higher odds of IPV. In all three sites, high controlling behaviour by partners was associated with greater increased odds of violence. In Juba, forced marriage was positively associated with IPV (COR = 7.7 95%CI = 2.3–23.6.)

In Rumbek and Juba, living in a polygamous marriage, and bride price were also associated with nearly a two-fold increase in the odds of IPV. In the Juba PoCs, having been married as a result of abduction, pregnancy or rape, and for economic reasons was associated with greater risk of IPV, whereas having married for love was protective. Finally, experiences of conflict were associated with IPV in all sites. In all three sites, having experienced rape or sexual exploitation increased the odds IPV, whereas in Rumbek and PoCs, attacks on villages and any traumatic event related to conflict were also associated with IPV. In Rumbek experiencing rape or attempted rape increased the odds of IPV by more than ten times (COR = 10.2, 95%CI = 5.1 = 20.4). In Rumbek, displaced due to conflict was also a risk factor, but not in PoCs, where all respondents had been displaced. All independent variables used in the site-specific analysis were significant in the pooled data set (Model 1), controlling for location. In the pooled multivariate model (Model 2), partners’ controlling behaviours, living in a polygamous marriage, or marriage due to abduction, pregnancy or rape (AOR = 2.0, 95%CI = 1.3–3.0), the belief that it is a woman’s obligation to have sex whenever their partner wants to (AOR = 1.6, 95%CI-1.2 = 2.0), and conflict related experiences of rape/attempted rape (AOR = 2.8, 95%CI-2.0 = 3.9), sexual exploitation (AOR = 2.2, 95%CI-1.5 = 3.2), or traumatic events (AOR = 1.8, 95%CI-1.4 = 2.3) were associated with increased risk of IPV, after controlling for age and location.

## Discussion

This is the first study to measure the prevalence and risk factors for different forms of violence against women and girls in South Sudan. A particular strength of the study is that it explored the effects of different forms and periods of conflict in relation to both IPV and NPSV. It also addresses the role of restrictive gender norms and practices, including polygamy, bride price, and child marriage and their influence on the risk of both IPV and NPSV. To our knowledge, this is the first study to address the interaction between conflict related sexual violence and other forms of VAWG.

NPSV was more than four times the global average in all three sites, although the perpetrators varied considerably. In Juba City, women and girls were more likely to have suffered sexual assault at the hands of family members or acquaintances, or by strangers (as in criminal gangs), and most of the assaults were not directly related to conflict. In contrast, the great majority of women and girls who were raped in the other two sites were directly related to conflict: intercommunal conflict and cattle raids, in the case of Rumbek, and in the case of the residents of the Juba PoC sites, sexual assault occurred in the context of attacks on villages, displacement due to the 2013 Crisis, and the daily dangers incurred in survival, such as leaving the sites to search for food or firewood.

Despite the catastrophic levels of non-partner sexual assault suffered by South Sudanese women, the greatest risk of physical and sexual violence came from their intimate partners. Over two thirds of women in all three sites had experienced physical and/or sexual violence IPV during their lifetimes. This is more than double the global average for IPV. The high frequency of IPV during the last 12 months indicates that violence starts early in a women’s life and lasts a lifetime.

A strength of the study is that it includes settings with different social and demographic characteristics, as well as different types of conflict. The study showed that both IPV and non-partner sexual violence are contextually specific, and the prevalence, characteristics, and risk factors may vary greatly among sites, even within the same country. However, the risk of IPV was associated across all sites with traditional attitudes towards gender and violence, and patriarchal practices such as polygamy. Conflict in all forms, but particularly conflict related sexual violence and exploitation greatly increased the risk of IPV.

The multivariate analysis measured the odds of being beaten or raped for women who endorse traditional attitudes around gender and violence against women. Although these findings are suggestive of the role of restrictive gender norms as drivers of both partner and non-partner violence, they should be interpreted with some caution. The measurement of social norms is a complex endeavour and beyond the scope of our study [24, 25]. However, indicators measuring individual or aggregate attitudes towards violence and gender equality have been used in multiple studies as a proxy measurement for social norms and their association with the risk of experiencing violence for women [26, 27] and for perpetrating violence, in the case of men [28]. The South Sudan study measured attitudes on gender and violence among both women and men, and the attitudes of men were similar to those of women in almost all instances [1]. It is likely that this association represents the increased risk of violence for women who live in communities where violence is tolerated or encouraged, as opposed to increased risk for individual women according to their attitudes. This is consistent with our own conceptual framework [20], and a broad international evidence base that has found restrictive gender norms to be among the strongest predictors of societies with a high prevalence of VAWG [26, 29–32].

Our conclusions are also consistent with findings from the qualitative research carried out as part of the same study. Interviews and focus groups discussion with both men and women indicate that gender inequitable norms, such as the view that women should be subordinate to men, and deserve to be beaten under some conditions, are extremely common, and contribute to the high prevalence of violence. This was reinforced by a traditional chief in Juba who explained, “It is common in our custom to beat a woman when she has made a mistake—not to the extent of killing her completely, but to discipline her” [1]. Practices such as polygamy, wife inheritance, and bride price, contribute to the view that women are the property of their husbands, and can be chastised if they transgress the boundaries of the established norms [13, 18, 33].

The qualitative findings also indicate that the economic crisis resulting from conflict has contributed to forced and child marriage, due to the loss of cattle wealth. Many families marry their daughters as a strategy to regain their wealth. In some settings, particularly in Rumbek, the loss of cattle wealth has led to an increase in cattle raids and abduction of girls by men who do not have enough cattle to pay bride price for marriage. It is also a key driver of sexual exploitation and abuse, particularly in the PoC sites, where the population is almost entirely dependent on humanitarian aid, and abuses were reportedly committed frequently by both humanitarian actors and community leaders.

A strong association was found between sexual violence by a non-partner and intimate partner violence. Because this was a cross-sectional survey, it is not possible to determine which type of violence led to the other. However, qualitative evidence from South Sudan and elsewhere suggest that married women who are raped during conflict are at high risk of violence if their partners discover the abuse. For an unmarried girl In South Sudan, where the amount of bride price is often dependent on the perceived “purity” of the girl, sexual violence is an even greater catastrophe. She may be considered “spoilt” and “unmarriageable,” hence the view that such a girl’s only option is to marry her abuser. This serves as an incentive for young men to rape girls, in order to marry them without paying bride price. If a married woman is raped, even during an attack on her village, it may be viewed as an act of adultery by her husband and the community. According to a female informant, “In case the husband heard [about the rape] and asked the wife she will totally refuse to tell the husband because he might claim the rapist knew her and it was an agreement. Then he will divorce the wife” [1].

There are several limitations of the study which may influence the findings. Firstly, the survey was only conducted in three sites, and therefore cannot be considered to represent the whole country. However, the estimates for 12-month IPV are in line with a recent study that collected national figures on current IPV [34], so it is likely that these figures are similar to other parts of South Sudan. Also, because the data collection in Juba was disrupted midway through, some bias may have been introduced into the Juba sample. Finally, as mentioned earlier, due to the cross-sectional design, it is not possible to infer causality, therefore findings should be interpreted with caution.

## Conclusion

Our findings paint a devastating portrait of violence suffered by women and girls in South Sudan throughout their lives, both as a result of the decades of conflict that the country has endured, but also due to the patriarchal norms and practices that treat women and young girls, as property that can be exchanged for other forms of property, such as cattle, and whose ownership infers absolute power over their reproductive choices, their ability to study and work, and their physical and sexual integrity.

Whilst the numerous UN Security Council Resolutions on Women, Peace, and Security and Sexual Violence in Conflict [35–37], have focused the world’s attention on conflict-related, non-partner sexual violence, this research adds a new dimension to the picture of VAWG in South Sudan–that women and girls experience multiple and compounding forms of violence that are exacerbated during times of conflict. Our findings contribute to the growing evidence that conflict-related sexual violence is not the only, or even the most common form of violence that women and girls are subjected to in the context of conflict and humanitarian crises [7, 11, 20]. These findings point to the need to expand conventional conceptions of conflict-related violence, as limited to sexual violence by armed actors, and embrace a more holistic definition that contextualizes sexual violence in relation to other forms of violence that also have a profound impact on their lives [20].

As South Sudan enters a precarious transitional phase of peace-building and state-building, it will be important to consider the range of women’s rights concerns, including VAWG, in the efforts to achieve sustainable positive peace for all. A practical recommendation emerging from these findings is that IPV should explicitly be included, as a human rights concern warranting specialised, targeted attention and programming, in donor and international humanitarian strategies, decision-making, and funding. Particular urgency should be given to addressing the needs of adolescent girls, who are frequently overlooked by current humanitarian approaches. Finally, although emergency provision of compassionate care for survivors of all forms of VAWG is critical, long-term strategies and funding must also be in place to address gender inequality and women’s economic empowerment, support local women’s groups, and ensure that the voices of women and girls are included in all aspects of the peace-building and state-building process.

## Supporting information

S1 File (PDF)

S1 Table (XLSX)
